# Recovery of Lignins with Potent Antioxidant Properties from Shells of Edible Nuts by a Green Ball Milling/Deep Eutectic Solvent (DES)-Based Protocol

**DOI:** 10.3390/antiox11101860

**Published:** 2022-09-21

**Authors:** Rita Argenziano, Federica Moccia, Rodolfo Esposito, Gerardino D’Errico, Lucia Panzella, Alessandra Napolitano

**Affiliations:** Department of Chemical Sciences, University of Naples “Federico II”, Via Cintia 4, I-80126 Naples, Italy

**Keywords:** lignin, antioxidant, waste valorization, deep eutectic solvents, nut shells, phenolic compounds, ball milling, ellagic acid, electron paramagnetic resonance

## Abstract

Lignins are phenolic polymers endowed with potent antioxidant properties that are finding increasing applications in a variety of fields. Consequently, there is a growing need for easily available and sustainable sources, as well as for green extraction methodologies of these compounds. Herein, a ball milling/deep eutectic solvent (DES)-based treatment is reported as an efficient strategy for the recovery of antioxidant lignins from the shells of edible nuts, namely chestnuts, hazelnuts, peanuts, pecan nuts, and pistachios. In particular, preliminarily ball-milled shells were treated with 1:2 mol/mol choline chloride:lactic acid at 120 °C for 24 h, and the extracted material was recovered in 19–27% *w*/*w* yields after precipitation by the addition of 0.01 M HCl. Extensive spectroscopic and chromatographic analysis allowed for confirmation that the main phenolic constituents present in the shell extracts were lignins, accompanied by small amounts (0.9% *w*/*w*) of ellagic acid, in the case of chestnut shells. The recovered samples exhibited very promising antioxidant properties, particularly in the 2,2-diphenyl-1-picrylhydrazyl (DPPH) assay (EC_50_ values ranging from 0.03 to 0.19 mg/mL). These results open new perspectives for the valorization of nut shells as green sources of lignins for applications as antioxidants, e.g., in the biomedical, food, and/or cosmetic sector.

## 1. Introduction 

Natural phenolic polymers from plant sources have been emerging in the field of material sciences as versatile, stable, and multifunctional additives [1,2,3]. A prominent role in this context is played by lignin, a heterogeneous polymer derived from peroxidase- or laccase-mediated oxidation of coniferyl, sinapyl, and *p*-coumaryl alcohol. Lignins are endowed with potent antioxidant and other functional properties that have prompted their applications in photocatalysis, stimuli-responsive materials, food packaging, wound healing, drug delivery, and tissue regeneration [4,5,6,7,8,9,10,11]. Given also the increasing interest in environmentally-friendly production processes, several studies have been directed to the exploitation of novel, easily available, and sustainable sources of these compounds, such as byproducts and wastes from the agri-food industry. Among these, shells from edible nuts that are produced in very large amounts such as peanut (11 million tons per year) [12], pecan nut (>420,000 tons per year) [13], hazelnut (>350,000 tons per year) [14], pistachio (>335,000 tons per year) [15], and chestnut (18,000 tons per year) [16,17] shells represent a most valuable alternative. Particular attention has been devoted in the past few years to green extraction methodologies, allowing for the efficient recovery of functional compounds such as phenols from natural sources through, e.g., the use of solvents with a low environmental impact. In this context, deep eutectic solvents (DESs) are emerging as eco-friendly and non-toxic media for application in the extraction not only of low-molecular-weight phenolic compounds, but also of phenolic polymers including lignin [18,19,20,21,22]. DESs are easily prepared by mixing, at a suitable temperature, a hydrogen bond acceptor (HBA) and a hydrogen bond donor (HBD), resulting in the formation of a eutectic mixture characterized by a melting point lower than that of the individual constituents. The ability of DESs to form hydrogen bonds or to donate and accept protons confers them good dissolution properties toward phenolic compounds, as recently explored in the case of agri-food wastes. A remarkable example is represented by 1:2 mol/mol chlorine chloride/lactic acid (ChCl:LA2), which has been reported as one of the most efficient solvents for lignin recovery [23,24,25,26].

Based on these observations, we report herein the exploitation of ChCl:LA2 for the recovery of lignin from peanut, pecan nut, hazelnut, chestnut, and pistachio shells. The recovered samples were characterized by electron paramagnetic resonance (EPR), attenuated total reflectance (ATR)-FTIR, ^1^H NMR, and UV-Vis spectroscopy as well as by chemical degradation experiments. The antioxidant properties of the samples were also determined by widely used chemical assays. 

## 2. Materials and Methods

### 2.1. General Experimental Methods

Pistachios, chestnuts, peanuts, hazelnuts, and pecan nuts were purchased at a local supermarket and shells were recovered by hand peeling. Benzyl mercaptan, 30% *w*/*v* hydrogen peroxide, 2,2-diphenyl-1-picrylhydrazyl (DPPH), 2,4,6-tris(2-pirydyl)-s-triazine (TPTZ), (±)-6-hydroxy-2,5,7,8-tetramethylchromane-2-carboxylic acid (Trolox), Folin–Ciocalteu reagent, ellagic acid, gallic acid, iron (III) chloride, syringic acid, vanillic acid, and vanillin were obtained from Sigma-Aldrich (Milan, Italy).

UV-Vis spectra were recorded using a Jasco (Cremella, Lecco, Italy) V-730 spectrophotometer.

ATR-FTIR spectra were recorded as an average of 128 scans in the range 4000−450 cm^−1^ (4 cm^−1^ resolution) on a Nicolet 5700 Thermo Fisher Scientific (Milan, Italy) instrument. 

^1^H NMR spectra were recorded in DMSO-*d*_6_ at 400 MHz on a Bruker (Milan, Italy) instrument.

HPLC analyses were performed on an instrument equipped with a UV-Vis detector (Agilent, Cernusco sul Naviglio, Milan, Italy); a Phenomenex (Castel Maggiore, Bologna, Italy) Sphereclone ODS column (250 × 4.60 mm, 5 µm) was used, and a gradient elution was performed using 0.1% formic acid (solvent A)/methanol (solvent B): 5% B, 0–10 min; from 5 to 80% B, 10–47.5 min. The flow rate was 1.0 mL/min, and the detection wavelength was 254 nm. 

LC-MS analysis were run on an Agilent (Cernusco sul Naviglio, Milan, Italy) LC-MS ESI-TOF 1260/6230DA instrument operating in positive ionization mode. The following conditions were adopted: capillary voltage 3500 V; drying gas (nitrogen) 5 L/min, 325 °C; fragmentor voltage 175 V; nebulizer pressure 35 psig. An Agilent (Cernusco sul Naviglio, Milan, Italy) Eclipse Plus ODS column (150 × 4.6 mm, 5 µm) was used, with the same eluant as above at a flow rate of 0.4 mL/min. 

Electron paramagnetic resonance (EPR) measurements were performed using a Bruker (Milan, Italy) Elexys E-500 spectrometer equipped with a superhigh sensitivity probe head. The samples were transferred in weighed amounts to a flame-sealed glass capillaries, which were then coaxially inserted in a 4 mm quartz sample tube. Spectra were recorded at room temperature under the following instrumental settings: sweep width 140 G; resolution 1024 points; scan time 20.97 s. The amplitude of the field modulation (1.0 G) was preventively checked to be low enough to avoid detectable signal overmodulation. To avoid microwave saturation of the resonance absorption curve, the microwave power was also optimized. A total of 16 scans were accumulated in order to improve the signal-to-noise ratio. For power saturation experiments, the microwave power was gradually incremented from 0.02 to 164 mW. The g value and the spin density were evaluated using Mn^2+^-doped MgO [27] as an internal standard. Since sample hydration was not controlled during the measurements, spin density values must be considered as order of magnitude estimates [28].

### 2.2. Pretreatment of Nut Shells 

Shells were manually broken into small pieces and roughly shredded using a common blender. The resulting powders were then finely minced by treatment in a Fritsch Pulverisette 23 ball mill (Emme 3, Lainate, Milan, Italy) for 15 min at 50 osc/s. 

### 2.3. DES Preparation 

A literature-reported procedure was followed [29]. Briefly, choline chloride and lactic acid were mixed at 1:2 mol/mol ratio and heated at 50 °C under stirring until a homogeneous liquid was formed. The DES thus obtained, indicated as ChCl:LA2, was stored in the dark at ambient temperature. No crystal precipitation was observed over the period of use.

### 2.4. Treatment of Nut Shells with DES 

A previously reported procedure was followed, with slight modifications [23]. Briefly, 10 g of each shell were added to 100 g of ChCl:LA2 containing 20% *w*/*w* water, and the mixture was taken under stirring in a Pyrex glass bottle at 120 °C for 24 h. After cooling at room temperature, the mixture was centrifuged (7000 rpm, 4 °C, 10 min) and the solid residue and supernatant were separated. Subsequently, the solid residue was washed three times with 50 mL of ethanol (7000 rpm, 4 °C, 10 min) and the collected washings were dried using a rotary evaporator. The obtained residue was added to the supernatant collected from the initial centrifugation, which was then poured into 1 L of 0.01 M HCl and kept at 4 °C until the precipitation of a brown solid was observed. The precipitate was then recovered by centrifugation (7000 rpm, 10 min, 4 °C), washed twice with 0.01 M HCl and once with distilled water, and lyophilized.

### 2.5. DPPH Assay 

Samples were added (final dose 0.02−0.4 mg/mL) to a 0.2 mM DPPH solution in ethanol [30,31]. After stirring at room temperature for 10 min, the absorbance of the solution at 515 nm was measured. Trolox was used as a reference antioxidant. Experiments were run in triplicate. 

### 2.6. Ferric Reducing/Antioxidant Power (FRAP) Assay 

Samples were added (final dose 0.025–0.2 mg/mL) to a solution of FeCl_3_ (1.7 mM) and TPTZ (0.83 mM) in 0.3 M acetate buffer (pH 3.6) [32]. After stirring at room temperature for 10 min, the absorbance of the solution at 593 nm was measured. Results were expressed as Trolox equivalents (eqs). Experiments were run in triplicate.

### 2.7. Total Phenolic Content (TPC) Assay 

Samples were added at a final dose of 0.0125−0.4 mg/mL to a mixture of Folin–Ciocalteu reagent, 75 g/L Na_2_CO_3_, and water in a 1:3:14 *v*/*v*/*v* ratio [33]. After incubation for 30 min at 40 °C, the absorbance at 765 nm was measured. Gallic acid was used as a reference compound. Experiments were run in triplicate. 

### 2.8. Vanillin-HCl Assay 

Samples were added (final dose 0.0625–2 mg/mL) to 1 mL of 1% *w*/*v* vanillin solution in methanol. Subsequently, 1 mL of 9 M HCl was added, and the mixture was incubated at 30 °C for 10 min [34]. Finally, the absorbance at 500 nm was measured. Catechin was used as a reference compound. Experiments were run in triplicate. 

### 2.9. Alkaline Hydrogen Peroxide Degradation 

Each sample (10 mg) was suspended in 1mL of 1 M NaOH and treated with 50 µL of 30% hydrogen peroxide [35]. The mixture was kept under vigorous stirring at room temperature for 24 h and then treated with 5% *w*/*v* Na_2_S_2_O_5_, taken to pH 3 with 6 M HCl, filtered on a 0.45 µm PVDF filter, and analyzed using HPLC.

### 2.10. Acid Degradation

Each sample (50 mg) was placed in a Pyrex tube, followed by 5 mL of 4 M HCl. The mixture was vortexed for 1 min and then incubated for 24 h in an oven at 90 °C [36]. After cooling to room temperature, the pH of the mixture was adjusted to 2.5 by addition of 6 M NaOH. The mixture was centrifuged at 7000 rpm for 10 min, the supernatant was recovered, taken to 10 mL by addition of water, and analyzed by HPLC after filtration on a 0.45 µm PVDF filter, whereas the solid residue was dissolved in 10 mL of DMSO/methanol 1:1 *v*/*v* and then analyzed by HPLC as well.

### 2.11. Thiolysis 

Each sample (8 mg) was treated in methanol (2 mL) with 50 μL of benzyl mercaptan and 20 μL of 37% HCl at 40 °C under stirring [37]. After 1 h, the mixture was diluted in 1:1 *v*/*v* methanol/water (5 mL) and analyzed by HPLC. 

## 3. Results and Discussion

### 3.1. Lignin Extraction from Nut Shells

Based on the previously reported results [23], a protocol based on the use of ChCl:LA2 as DES was adopted for the recovery of lignin from the selected nut shells. Before extraction, the latter were finely minced with the aid of a ball mill with the aim of improving the extraction yields by both increasing the surface area and cleaving glycosidic bonds and β-O-4 bonds of lignin components [38,39,40,41], also resulting in the liberation of phenolic hydroxyl groups and hence, boosting of the antioxidant properties. Subsequent extraction was performed at 120 °C for 24 h, allowing for the recovery lignins in the form of brown/black powders by simple precipitation, centrifugation, and lyophilization.

Table 1 reports the extraction and recovery yields obtained from the different nut shells, calculated with respect to the amount of starting or DES-dissolved material, respectively. The highest values were observed in the case of hazelnut and pistachio shells. In particular, the hazelnut shell lignin yields were higher than those reported in literature using different extraction procedures [42,43], whereas no data have apparently been reported for pistachio and pecan nut shells. A ca. 36% *w*/*w* yield has been recently reported for peanut shell lignin using 6:4 *v*/*v* ethanol/water as solvent at 180 °C for 100 min [44]. Though the yield is comparable to that obtained here, it should be noted that in the present paper a higher, and hence more cost effective and environmentally friendly, solid-to-solvent ratio was adopted. Chestnut shells exhibited the lowest extraction/recovery yields, although comparable to those reported in literature using different solvents [45]. In any case, it is noteworthy that to the best of our knowledge, there are no data available in the literature on the combined use of ball milling and DES for lignin recovery from edible nut shells.

### 3.2. Structural Characterization of Nut Shell-Derived Lignins 

#### 3.2.1. Spectroscopic Analysis 

**Figure 1:**
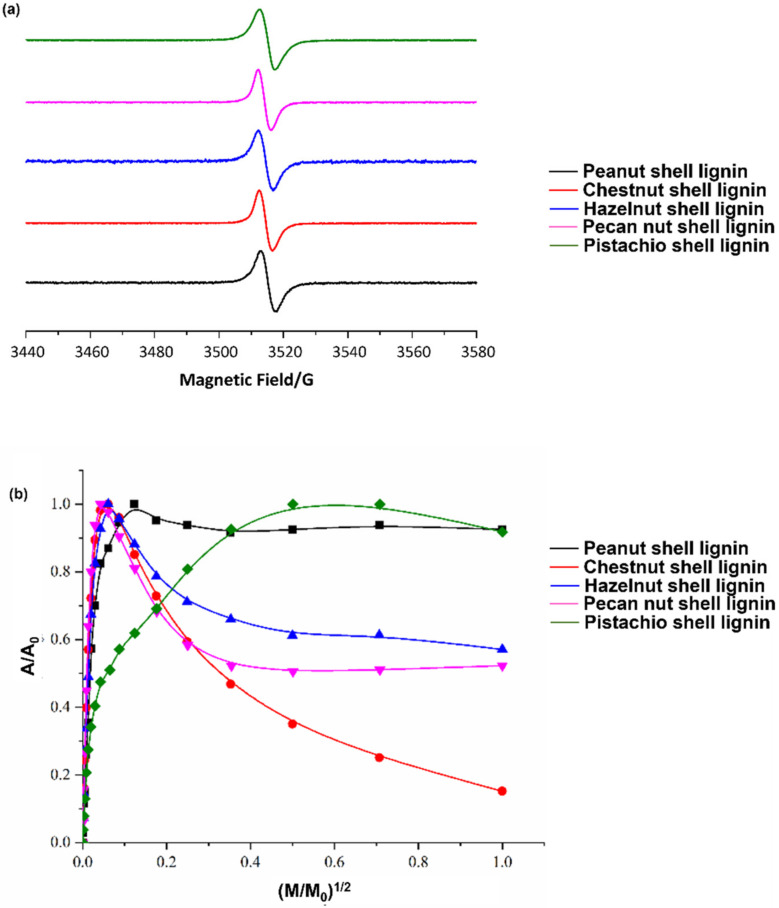
(**a**) Solid-state EPR spectra and (**b**) power saturation profiles of lignins recovered from the different nut shells.

For the structural characterization of the recovered lignins, EPR spectroscopy was initially exploited, since it has been widely proven to be a sensitive and specific technique for the identification of phenolic polymers, which are known to be characterized by the presence of intrinsic free radical centers [1,23,46,47,48]. The EPR spectra of all samples showed a singlet (Figure 1a and Figure S1) at g values typical of other lignin-rich samples and consistent with the presence of carbon-centered radicals (Table 2) [23,49]. Despite the fact that EPR signals could appear featureless, a quantification of their intensity and a thorough analysis of their line shape (in terms of signal broadness and Gaussian or Lorentzian line shape) furnish a wealth of information on the nature, molecular structure, and supramolecular organization of the radical species present in a sample [46]. Indeed, while the starting nut shells exhibited apparently asymmetric spectra (Figure S1), likely as the result of the superposition of signals arising from different radical species, all the recovered lignins exhibited a symmetric signal (Figure 1a) with an intermediate line shape between the derivatives of the Gaussian and Lorentzian functions (Table 1). Moreover, recovered lignins showed higher spin density and lower signal amplitude (ΔB) values with respect to the starting material (Table 1), highlighting the efficacy of DES-based extraction in providing samples enriched in the lignin component and characterized by a more homogenous free radical population and a relatively higher extent of π-electron delocalization [23,46]. In addition, in the case of chestnut, hazelnut, and pecan nut shell lignins, the normalized power saturation curves (Figure 1b) showed a well-pronounced intensity decrease at high microwave power, indicating a strong homogeneous relaxation behavior and confirming a low degree of variety in the free radical population of the DES-recovered samples [23].

ATR-FTIR spectra evidenced for all the DES-recovered samples the presence of two sharp peaks in the 2950–2850 cm^−1^ region, typically associated to the aromatic C−H bond stretching vibration of lignins (Figure S2) [1,50].

**Figure 2:**
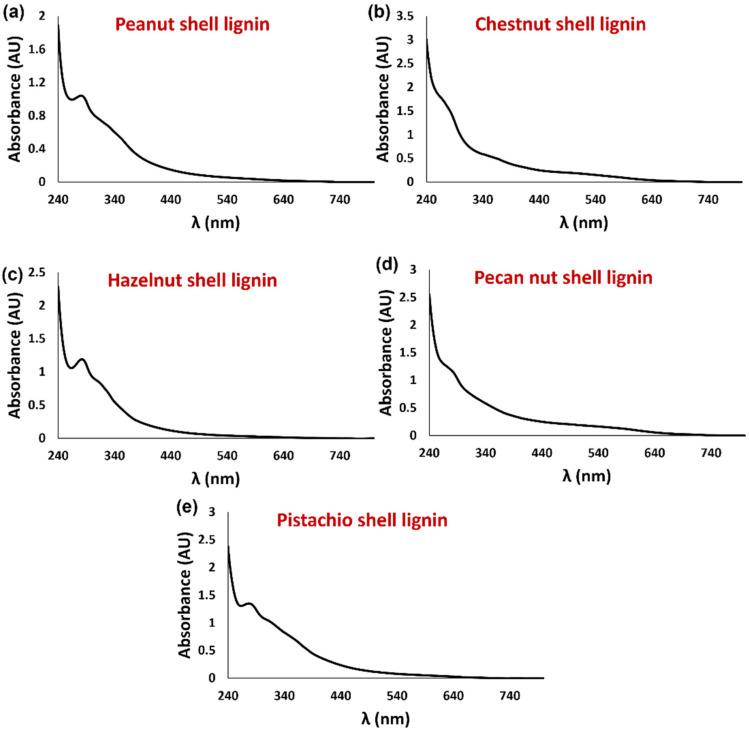
UV-Vis spectra of the DMSO-soluble fraction of (**a**) peanut, (**b**) chestnut, (**c**) hazelnut, (**d**) pecan nut, and (**e**) pistachio shell lignins.

The UV-Vis spectra of the DMSO-soluble fraction of the recovered lignins were also recorded (Figure 2). DMSO was chosen as the solvent based on its ability to dissolve a wide range of most polar and non-polar natural phenolic compounds. All the samples exhibited a broadband absorption in the range 240–640 nm, with a hint of absorption at around 280 and 310 nm typical of lignin moieties [51,52], which was particularly evident for the samples deriving from peanut, hazelnut, and pistachio shells. 

Finally, the recovered lignins were analyzed by ^1^H NMR spectrometry (Figures S3–S5) using DMSO-*d*_6_ as solvent. For all the samples, a very broad complex signal at 6.0–7.4 ppm, indicative of the presence of a heterogeneous phenolic polymer such as lignin [23,53], was observed. Notably, in the case of chestnut shell lignin, a singlet at 7.48 ppm was also observed, likely due to the presence of a small amount of ellagic acid [54], in line with what previously reported for DES-based extractions of chestnut shells [55,56]. Chestnut shell lignin, and to a lesser extent pecan nut shell lignin, were also the only samples to exhibit a well-detectable broad signal in the range of 8.5–9.5 ppm, likely due to the presence of a significant number of phenolic OH groups.

#### 3.2.2. Chromatographic Analysis 

**Figure 3:**
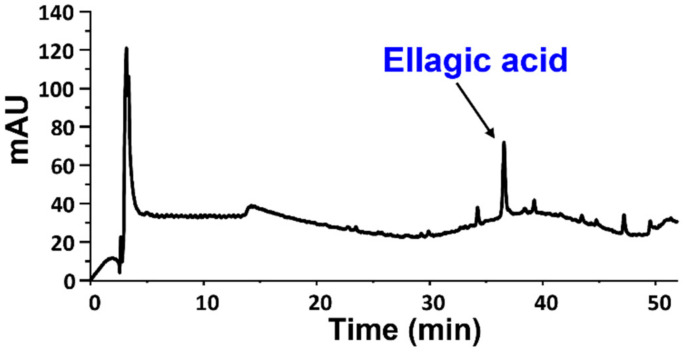
HPLC profile of the DMSO-soluble fraction of chestnut shell lignin.

To acquire additional data on the composition of the DES-recovered samples from the different nut shells, the DMSO-soluble fractions were analyzed also by HPLC after proper dilution in methanol. In agreement with what was observed by ^1^H NMR analysis, the elutographic profiles showed no significant peaks for any samples, suggesting the absence of phenolic compounds with a low molecular weight. The only exception was the chestnut shell-derived sample, showing a peak around 36 min (Figure 3), which was attributed to ellagic acid by LC-MS analysis and comparison of the chromatographic properties with those of an authentic standard. This compound was found to be present at a ca. 0.9% *w*/*w* amount in the chestnut shell DES-extracted sample.

In order to obtain more information on the non-cromatographable components of the different samples, chemical degradation treatments commonly used for the qualitative and quantitative analysis of phenolic polymers were performed. These involved thiolysis, acid degradation, and alkaline hydrogen peroxide degradation. The first is commonly used to analyse condensed tannins [57], whereas acid degradation is employed to detect extractable and non-extractable ellagitannins in plant materials [36]. Alkaline hydrogen peroxide degradation enables the analysis of insoluble and structurally complex phenolic polymers such as melanin pigments and lignins, and is based on the identification of chromatographable, low-molecular weight markers deriving from the oxidative breakdown of the polymer [35]. 

HPLC analysis of the thiolysis mixtures showed the presence of some low-intensity peaks for all of the recovered lignins (not shown). However, the results of the spectrophotometric vanillin-HCl assay [34,57] indicated the absence of detectable amounts of condensed tannins in all the samples.

In the case of acid degradation mixtures, no products were detected for any samples, indicating the absence of significant amounts of hydrolyzable tannins in all of them. 

Finally, the HPLC profiles of the alkaline hydrogen peroxide degradation mixtures showed, among others, two main peaks eluted at 25.0 and 26.7 min (Figure S6), identified as vanillic and syringic acid, respectively, based on LC-MS analysis and on the comparison of the chromatographic properties with those of authentic standards. In particular, both these compounds, which are indicative of the presence of guaiacyl and syringyl units in the lignin samples, respectively, were found in the elutographic profiles of the degradation mixtures of hazelnut, peanut, and pistachio shell lignins. On the other hand, only small amounts of syringic acid were observed in chestnut shell lignin, whereas in the case of pecan nut shell lignin, only vanillic acid was observed.

Based on the results of both spectroscopic and chromatographic analysis, it can be concluded that the ball milling/DES-based extraction approach adopted in this study allowed for the acquisition of structurally diverse lignin-rich samples of good purity from all of the selected nut shells.

### 3.3. Antioxidant Properties of the Nut Shell-Derived Lignins 

In a last series of experiments, the recovered lignins were characterized for their antioxidant properties and TPC with commonly used spectrophotometric assays. 

**Figure 4:**
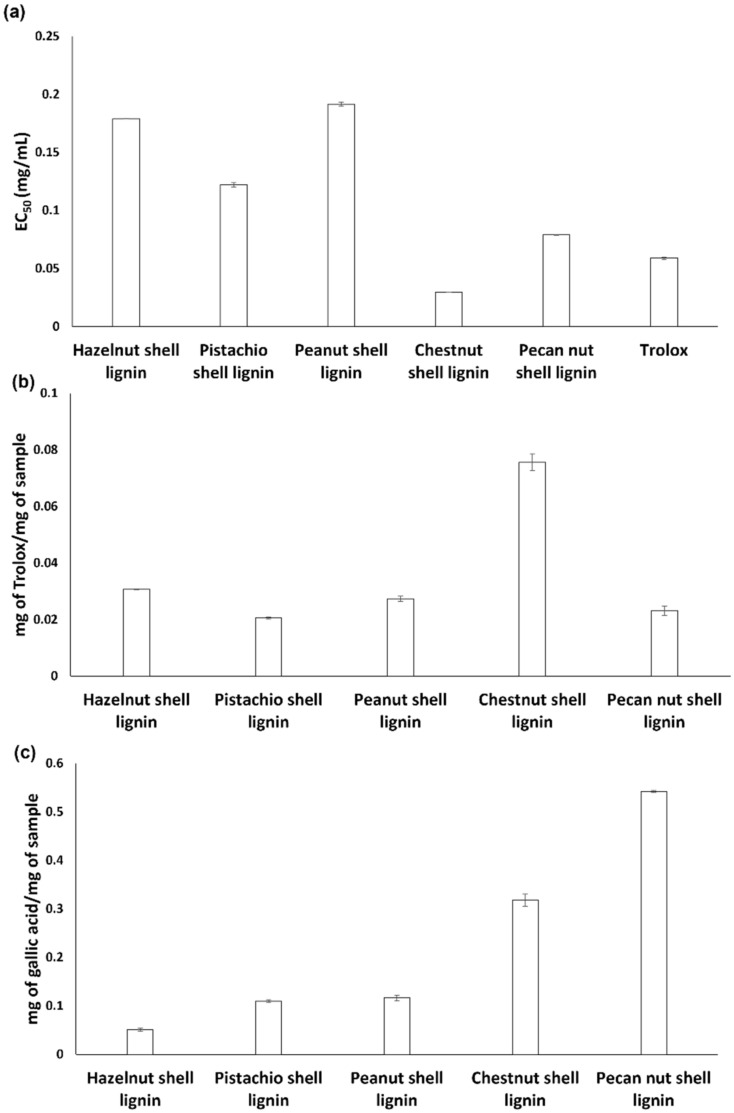
Antioxidant properties and TPC of nut shell-derived lignins. (**a**) DPPH assay; (**b**) FRAP assay; (**c**) Folin-Ciocalteu assay. Reported are the mean ± SD values from at least three experiments.

In the DPPH assay, chestnut shell lignin was the most active material, exhibiting an EC_50_ value lower than that determined for the reference antioxidant compound Trolox (Figure 4a). Good DPPH-reducing properties were also observed for pecan nut shell lignin, confirming the high efficiency of this agri-food byproduct as a source of antioxidant compounds [1,58]. On the other hand, peanut, hazelnut and pistachio shell lignins were characterized by EC_50_ values significantly higher than that of chestnut and pecan nut shell lignins. 

Chestnut shell lignin also exhibited the highest reducing properties in the FRAP assay, whereas all the other samples were characterized by comparable Trolox equivalent values (Figure 4b).

The superior antioxidant properties of chestnut shell lignin in both the DPPH and FRAP assay would be in line with the presence of the relatively high number of phenolic OH groups detected by ^1^H NMR analysis. It is also noteworthy that based on the amount of EA present in the chestnut shell lignin and the antioxidant properties reported for pure EA [23], this compound accounts for only 5–12% of the DPPH and Fe^3+^-reducing properties of the chestnut shell-derived sample, highlighting the real antioxidant effectiveness of chestnut lignin.

In agreement with the results of the antioxidant assays, the highest TPCs as determined by the Folin–Ciocalteu assay were observed for pecan nut shell and chestnut lignins (Figure 4c).

The discrepancy in the sample ranking emerging from the three assays is likely the result of the different properties monitored by each of them. Indeed, while the FRAP assay measures the electron transfer capacity of an antioxidant, the DPPH assay is a mixed-mode assay, as DPPH can be reduced through both an electron transfer and a hydrogen atom transfer mechanism [59]. On the other hand, the very high working pH required by the Folin–Ciocalteu assay may lead to an overestimation of the phenolic content and hence, of the antioxidant properties [60]. Of course, the relative solubility in the assay medium might also affect the antioxidant efficiency of the samples.

**Figure 5:**
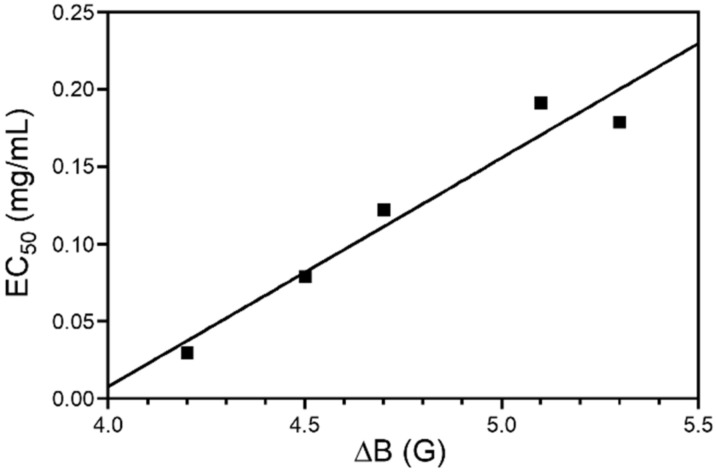
Correlation between antioxidant properties (expressed as EC_50_ values determined in the DPPH assay) and ΔB values (determined by EPR spectroscopy) of nut shell lignins (EC_50_ = (−0.58 ± 0.10) + (1.15 ± 0.02) ΔB, R^2^ = 0.94).

Notably, a remarkable direct linear correlation (R^2^ = 0.94) was found between the EC_50_ values from the DPPH assay and the ΔB values of the EPR spectra (Figure 5)**.** This is in agreement with what has been previously reported for synthetic, biomimetic phenolic polymers [46,61], and would highlight the importance of π-electron spin delocalization, one of the main factors driving EPR signal narrowing, as a structural determinant of the antioxidant properties of phenolic polymers, including lignin.

## 4. Conclusions

In conclusion, a green protocol for the recovery of lignins from the shells of edible nuts based on ball milling/DES extraction is reported. The proposed approach allowed for the recovery in good yields of lignin-rich samples of good purity, characterized by very efficient antioxidant properties, comparable or even higher than those of the reference antioxidant Trolox, particularly in the DPPH assay. This is likely the result of the efficacy of the ball milling pre-treatment and the acidic nature of the DES employed in inducing cleavage of glycosidic and β-O-4 bonds of the lignin components, resulting in a higher availability of antioxidant phenolic OH groups. Although the potential of chestnut and pecan nut shells as sources of antioxidant compounds is well documented, the results of this study highlight the possibility of exploiting other widely produced nut shells to recover lignin samples for application as antioxidant additives in the biomedical, food, and/or cosmetic sectors, from a green chemistry perspective. In addition, this work provides, for the first time, a comparative and systematic evaluation of both the antioxidant and structural properties of lignins recovered from edible nut shells, highlighting the importance of the structural features (as investigated by spectroscopic and chromatographic techniques) in determining the functional properties of natural, complex phenolic polymers.

## Figures and Tables

**Table 1:** Lignin extraction and recovery yields from selected nut shells.

Shell	Extraction Yield (% *w*/*w*_s_) ^1^	Recovery Yield (% *w*/*w*_d_) ^1^
Peanut	19	34
Chestnut	19	27
Hazelnut	25	36
Pecan nut	19	34
Pistachio	27	38

^1^*w*_s_: weight of starting material; *w*_d_: weight of the DES-dissolved material.

**Table 2:** EPR data for nut shells and related lignins.

Sample		Spin/g Concentration	ΔB (±0.2)	Gauss Fraction (±0.05) ^1^	g-Factor (±0.0003)
Peanut shell	Starting	2.6 × 10^16^	5.6	-	2.0038
	Lignin	2.2 × 10^17^	5.1	0.42	2.0030
Chestnut shell	Starting	1.6 × 10^16^	5.9	-	2.0031
	Lignin	3.5 × 10^17^	4.2	0.70	2.0031
Hazelnut shell	Starting	2.1 × 10^16^	7.0	-	2.0031
	Lignin	6.6 × 10^16^	5.3	0.21	2.0036
Pecan nut shell	Starting	1.4 × 10^16^	4.8	-	2.0026
	Lignin	4.6 × 10^17^	4.5	0.48	2.0030
Pistachio shell	Starting	5.9 × 10^15^	6.8	-	2.0037
	Lignin	2.5 × 10^17^	4.7	0.43	2.0027

^1^ For the spectra of the starting material showing the superposition of signals, the Gaussian fraction was not determined.

## Data Availability

All of the data is contained within the article.

## Supplementary Materials

The following supporting information can be downloaded at: https://www.mdpi.com/article/10.3390/antiox11101860/s1, Figure S1. Solid state EPR spectra of starting nut shells. Figure S2. ATR-FTIR spectra of (a) peanut, (b) chestnut, (c) hazelnut, (d) pecan nut and (e) pistachio shell lignins. Figure S3. ^1^H NMR spectra (DMSO-*d*_6_) of (a) peanut and (b) chestnut shell lignins. Figure S4. ^1^H NMR spectra (DMSO-*d*_6_) of (a) hazelnut and (b) pecan nut shell lignins. Figure S5. ^1^H NMR spectrum (DMSO-*d*_6_) of pistachio shell lignin. Figure S6. HPLC profiles of the alkaline hydrogen peroxide degradation mixtures of (a) peanut, (b) chestnut, (c) hazelnut, (d) pecan nut, and (e) pistachio shell lignins.
