# Supplementary material for: Recovery of Lignins with Potent Antioxidant Properties from Shells of Edible Nuts by a Green Ball Milling/Deep Eutectic Solvent (DES)-Based Protocol
Source: Antioxidants (Basel). 2022 Sep 21;11(10):1860. doi: 10.3390/antiox11101860 (PMC9598286; doi:10.3390/antiox11101860)
Supplement: Supplementary file 1 [file antioxidants-11-01860-s001.zip › antioxidants-1910505-supplementary.pdf]

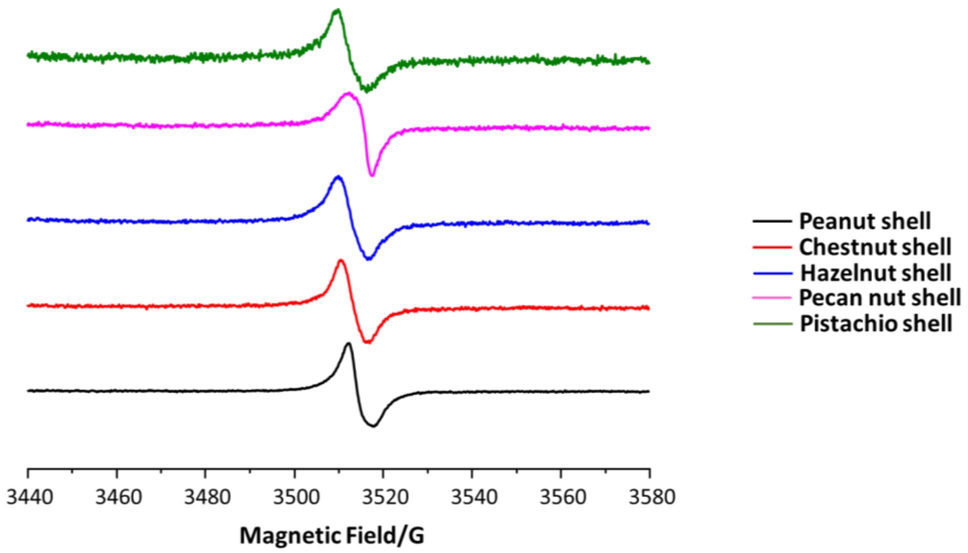

Figure S1. Solid state EPR spectra of starting nut shells.

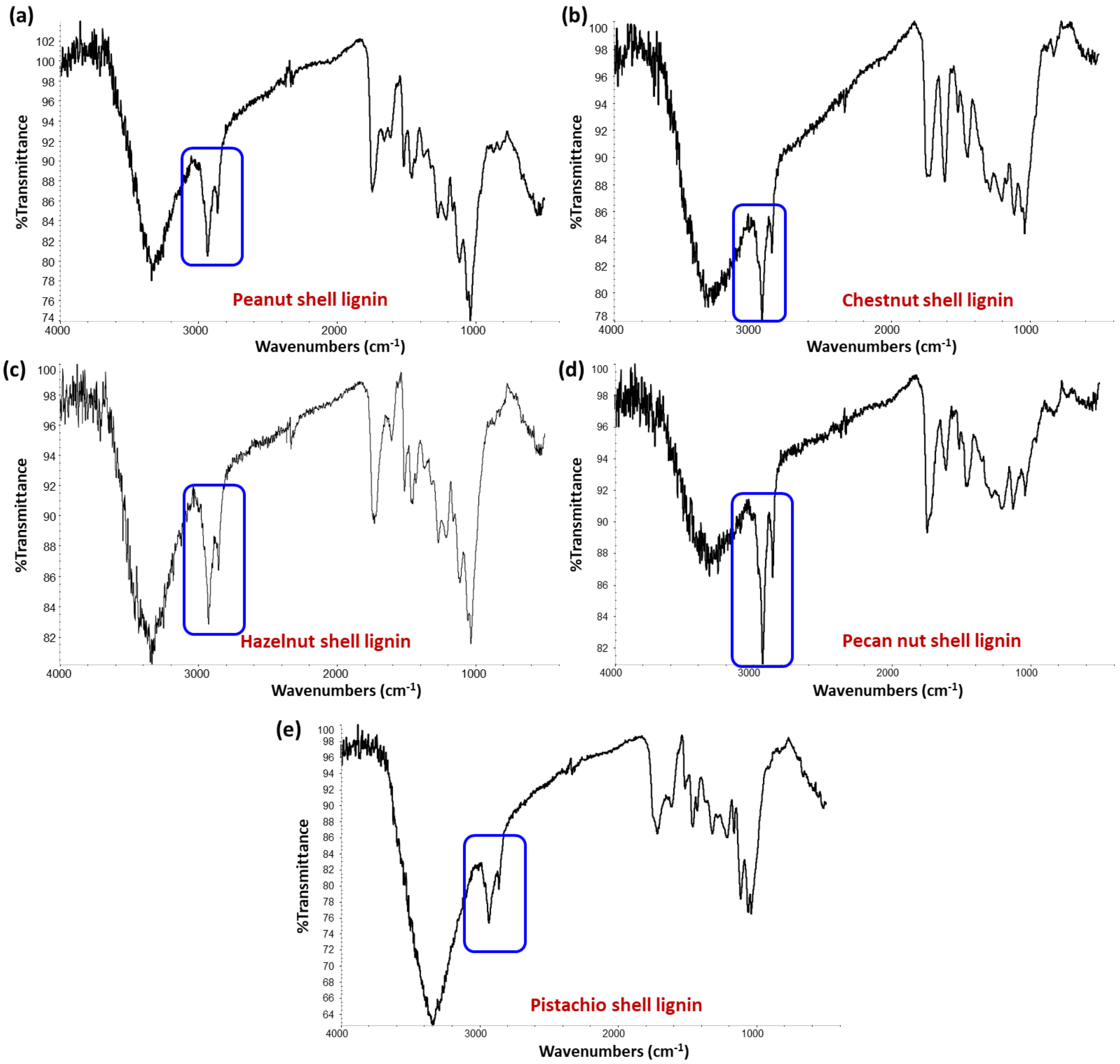

Figure S2. ATR-FTIR spectra of (**a**) peanut, (**b**) chestnut, (**c**) hazelnut, (**d**) pecan nut and (**e**) pistachio shell lignins. Highlighted are the bands associated to the aromatic C−H bond stretching vibration of lignins.

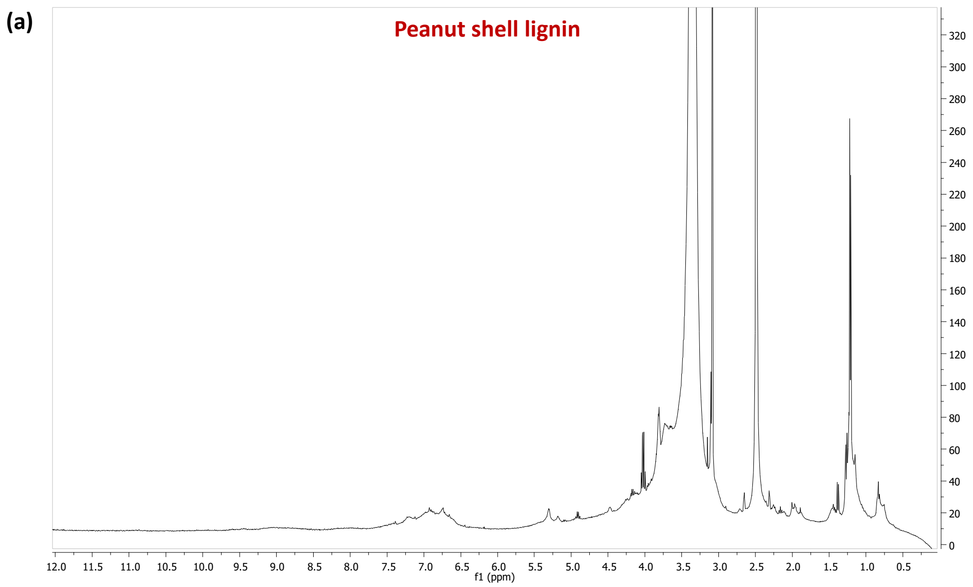

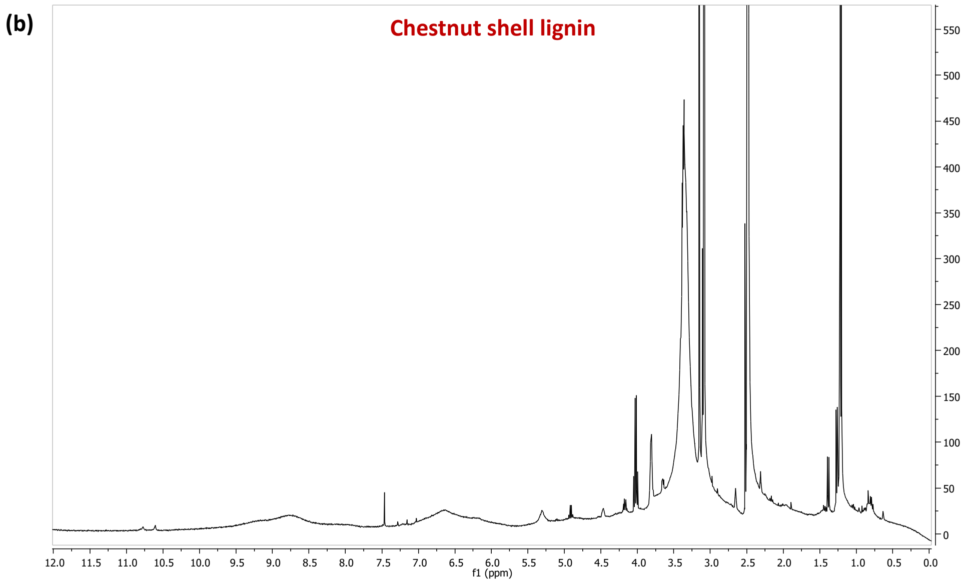

Figure S3. ^{1}H NMR spectra (DMSO-d_6) of (**a**) peanut and (**b**) chestnut shell lignins.

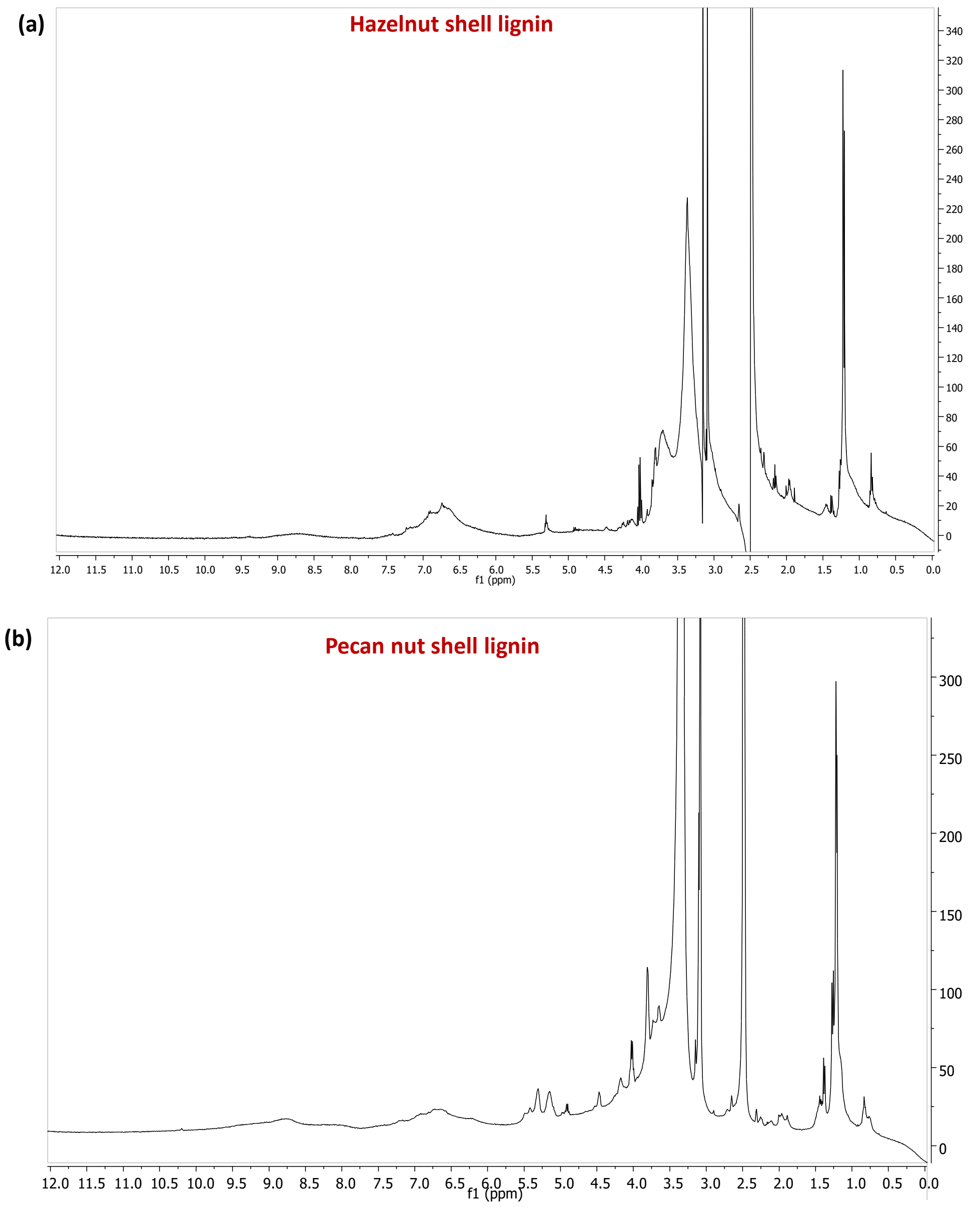

Figure S4. ^{1}H NMR spectra (DMSO-d_6) of (**a**) hazelnut and (**b**) pecan nut shell lignins.

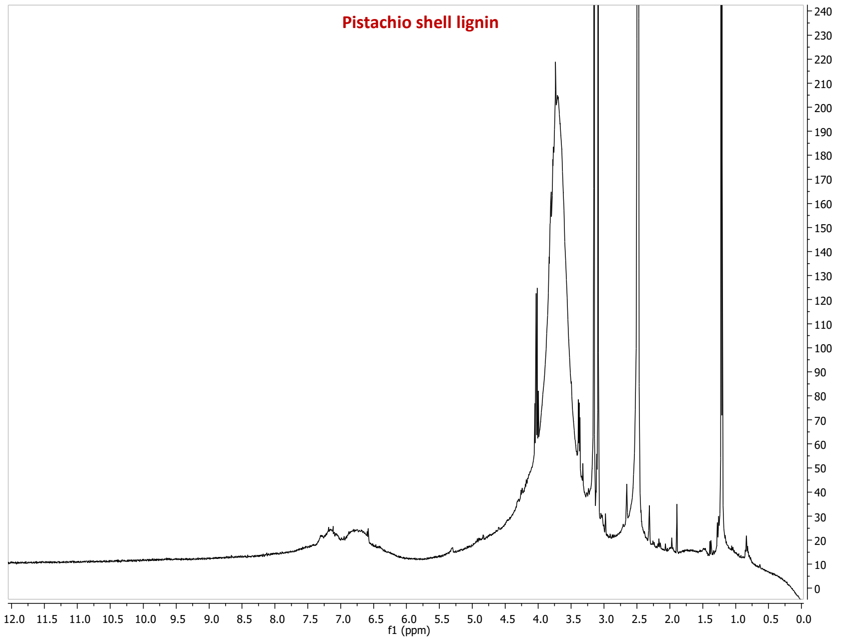

Figure S5. ^1H NMR spectrum (DMSO-d_6) of pistachio shell lignin.

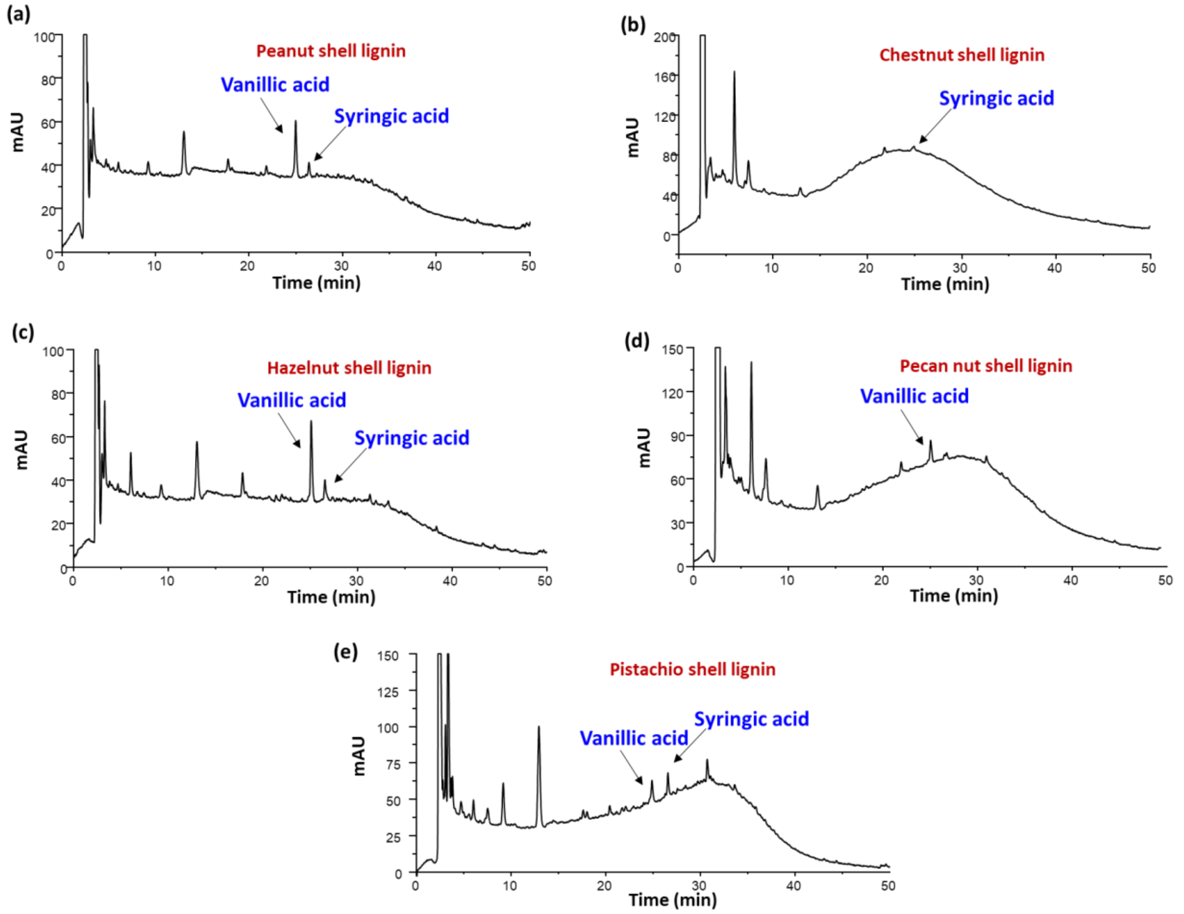

Figure S6. HPLC profiles of the alkaline hydrogen peroxide degradation mixtures of (**a**) peanut, (**b**) chestnut, (**c**) hazelnut, (**d**) pecan nut, and (**e**) pistachio shell lignins.
